# Digested Extracts from Laennec, on the Chest

**Published:** 1828-10

**Authors:** 


### Art. IV.—*Extracts from Laennec on Diseases of the Chest, supplementary to the preceding case.*

As the *unique* and valuable Treatise of M. Laennec, is by no means so extensively disseminated and profoundly studied by the physicians of the Western States, as it deserves to be; we shall collect from it, and reprint under distinct heads, his principal observations on the different læsions, which existed in the foregoing case.—Ed.

### 1.—EMPHYSEMA OF THE LUNGS.

The disease which I designate by this title is very little known, and has not hitherto been correctly described by any author. I for a long time thought it very uncommon, because I had observed only a few cases of it; but since I have made use of the stethoscope, I have verified its existence as well on the living as the dead subject, and am led to consider it as by no means unfrequent. I consider many cases of asthma, usually deemed nervous, as depending on this cause. The chief reason of this affection having been so completely overlooked is, that it is in some sort merely the *exageration* of the natural condition of the viscus.

In order that we may have a clear idea of the disease in question, it may be useful to advert to the natural organization of the pulmonary tissue. In examining, in a good light, the surface of the healthy lung, we can perceive, even with the naked eye, through the transparent pleura, which forms its covering,that the parenchyma of the viscus consists of an aggregate of small vesicles, irregularly spheriod or ovoid, filled with air, and separated from each other by opaque white partitions. These vesicles,—which, as seen on the surface of the lung, have the appearance of small transparent points,—are not all of one size. The largest are one third or one fourth part the size of a millet-seed. These are grouped in masses or lobules, which are circumscribed by partitions of condensed cellular substance, very thin, but yet thicker and more opaque than the partitions which separate the individual air-cells. These larger partitions traverse the lungs in all directions, and, consequently, in cutting each other at every possible angle, form, on the surface, figures of very various shapes—lozenges, squares, triangles, &c. It is along these partitions that the black pulmonary matter of which we have already spoken, is most plentifully deposited.

In emphysema of the lungs, the size of the vesicles is much increased, and is less uniform. The greater number equal, or exceed the size of millet-seed, while some attain the magnitude of hemp-seed, cherry-stones, or even French beans (haricot). These latter are probably produced by the reunion of several of the air-cells through rupture of the intermediate partitions; sometimes, however, they appear to arise from the simple enlargement of a single vesicle. The largest of these dilated cells are often in no respect prominent on the surface of the lung; sometimes they form a slight projection. In the latter case the structure of the lung acquires a striking resemblance to the vesicular lungs of the Linnæan Reptilia. Sometimes, though more rarely, we observe on the surface of the lung single vesicles, distended to the size of a cherry-stone or larger, quite prominent, exactly globular, and apparently pediculated. I say *apparently* pediculated, because on cutting into them we find that there is no real pedicle, but merely a constriction at the point where the cell begins to rise beyond the surface of the lung. The cavity of these dilated cells descends some little way into the substance of the viscus, and there its walls do not collapse, when cut, as in the projecting portion. At the bottom of this inferior portion of the cavity, we find small openings by which the dilated cell communicates with the adjoining ones, and with the bronchia. That these projecting vesicles are produced by the dilatation of an air-cell, and are not owing to the extravasation of air under the pleura, is proved, as well by the prolongation, just mentioned, of their cavity into the pulmonary substance, as by the circumstance that we cannot force the contained air, by pressure with the finger, to leave its place and to pass under the contiguous pleura,—as would be the case if it were extravasated.

As long as the parts continue in the state above described, the disease consists merely in an excessive, permanent, and unnatural distention of the air-cells, the air being still contained in its proper cavities; but when the distention becomes still more considerable, or takes place with greater rapidity, the air-cells are ruptured in certain points, and the surrounding cellular substance of the lung becomes distended by extravasated air, exactly in the same manner as in emphysema of the subcutaneous adipose membrane. In this case we find on the surface of the lung vesicles of an irregular form, which can be made to change their place by pressure with the finger. They vary in size from that of a hemp-seed to that of a walnut, or even an egg. Like the simply dilated cells, these vesicles contain nothing but air, which makes its escape on their being punctured with a pin. Sometimes the air, though truly *extravasated* under the pleura, cannot be displaced by pressure in the manner just mentioned. This happens when the extravasation is situated at the point of reunion of the partitions which divide the different groups of air-cells, as above mentioned. In this case the projection has usually a triangular shape, and is not very considerable.

I have never found this extravasated air penetrate, to any considerable extent, into the substance of these interlobular partitions, nor into the cellular substance which surrounds the larger blood-vessels and bronchial trunks; but I have seen the pulmonary substance in the interior of the lung lacerated by over-distention of the air-cells. These lacerated portions contain air, and sometimes also a small quantity of blood, either coagulated or loose; and the surrounding air-cells, which form the immediate walls of the excavation produced by the rupture, are observed to be loose, flabby, and without their natural globular figure.

The bronchial tubes, especially those of a small calibre, are sometimes very evidently dilated in those portions of the lung where the emphysema exists. This is easily proved by comparing the diseased and sound portions of the lungs. It is a thing to be expected; and it is, indeed, singular that it is not more common, since the cause which dilates the air-cells must act equally on the bronchia: it is, notwithstanding, very rare.

Emphysema may affect both lungs at the same time, one only, or a part of one or of both. In the latter case,—and indeed in any case, as long as there do not exist vesicles of considerable size on the superficies of the lungs,—it is easy to overlook the disease in the dead subject, and, as I have already said, I am convinced that this has often been done, not only by myself but by the best practical anatomists. For my own part I am convinced, that if we carefully examine the lungs of subjects who have long suffered from dyspnœa, from whatever cause, we shall almost always find more or fewer of the air-cells dilated. In lungs studded with tubercles, which presented no other symptom of emphysema, I have sometimes found two or three of the cells dilated to the size of a hemp-seed.

When the disease exists in a high degree, and occupies the whole of one or both lungs, one cannot help being struck with the appearance of the parts. The lungs seem as if confined in their natural cavity, and, when exposed, instead of collapsing as usual, they rise in some degree and project beyond the borders of the thorax. If we examine them in this state they feel firmer than natural, and it is more than usually difficult to flatter them. The crepitation they afford on pressure, or on being cut into, is less, and of a kind somewhat different; it is more like the sound produced by the slow escape of air from a pair of bellows; and the air makes its escape from the cells much quicker than in a healthy state of the organ. When we detach the lung, the crepitation is found to be still less perceptible, and the sensation conveyed by pressing the parts is very like that produced by handling a pillow of down. On placing an emphysematous lung in a vessel of water it sinks much less than a healthy lung; sometimes it floats on the surface with scarcely any obvious immersion. The pulmonary tissue is drier in a lung affected with emphysema, than in a healthy one; and it is unusual to find, even towards the root of the lungs, any trace of the common serous or sanguinous engorgements usually found after death. The contrary, however, is sometimes the case. When a single lung is affected, it becomes much more voluminous than that of the other side, so much so, indeed, as sometimes to press on one side the heart and mediastinum, and to cause an evident enlargement of the bony compages of that side of the chest.

From these observations it results, that Emphysema of the lungs consists essentially in the dilatation of the air-cells, and that the *extravasation* of the air on the surface of the lungs, constituting the larger and more prominent vesicles, is a posterior affection, and not necessarily connected with the disease in question. The latter species of lesion is, moreover, one of slight consequence compared with the dilatation of the cells, as we can hope for its removal by absorption, as in other similar cases; whilst we can hardly conceive in what manner either nature or art can remedy the other morbid derangement.—*Philadelphia Ed.*—pp. 82—86.

These observations I wish to confine to the emphysema from internal causes, without at all including the disease mentioned by surgical writers under this name, produced by the introduction of foreign bodies into the trachea, and which disease they describe as consisting in an extravasation of air into the cellular substance interposed between the air-cells. It is indeed true that such an accident, as well as violent exertions of the lungs, in childbirth, &c. can and do sometimes produce emphysema of the neck, chest, &c.; but such an affection can only arise from rupture of the bronchial tubes or air-cells: and I am disposed to believe that in no such case is there any actual penetration, by the extravasated air, of the interlobular cellular substance of the lungs themselves.—p. 88.

The general symptoms of this affection are rather equivocal. Dyspnœa being its most striking feature, it is one of the diseases usually confounded under the name of asthma. In it the respiration is habitually impeded, but is aggravated by occasional paroxysms which are quite irregular in their return and duration. Like dyspnœa from any other cause, it is further increased by the usual causes, such as indigestion, mental emotion, elevated situation, violent exercise, especially that of mounting, &c. It is unaccompanied by any fever, and the pulse is, for the most part, regular. When the affection exists in a high degree, the skin assumes a dirty aspect, with a bluish tint in some places, especially the lips. In all the cases I have seen there was a slight degree of habitual cough, with a very slight mucous expectoration. The complaint often exists from childhood, and does not seem materially to abridge the duration of life. Like other dyspnœas it frequently, in the end, gives rise to hypertrophia or dilatation of the heart.

When this disease occupies only one side, or exists much more in one lung than the other, this side is evidently enlarged, and the intercostal spaces wider. It also yields a more distinct sound on percussion. When both sides are equally affected, the chest yields a very distinct sound throughout, and presents a more rounded outline, both before and behind, than is natural in the sound state of that cavity.

The pathognomonic sign of this disease is furnished by a comparison of the indications derived from percussion and auscultation.—The respiratory murmur is inaudible over the greater part of the chest, and is very feeble in the parts where it is audible: at the same time a very distinct sound is produced by percussion. If the disease is not very severe, the sound of the respiration is still audible, but in a much less degree than the sound on percussion would lead us to expect. There is also heard, in the affected parts, an occasional slight sibilous rattle.

The single circumstance—of the absence of the noise of respiration in a chest, which sounds well on percussion,—is sufficient to distinguish emphysema of the lungs from any other disease of the chest except pulmonary catarrh and pneumo-thorax. We have already made some remarks on the distinction between the former disease and emphysema; and may repeat, that the general symptoms are sufficient to enable any one to discriminate them. The means of distinguishing emphysema from pneumo-thorax will be noticed under the account of the latter disease.

It is difficult to account for the absence of the sound of respiration, in a disease which consists essentially in dilatation of the air-cells, and in which, consequently, there exists more air than is usual in the lungs. The fact is, probably, accounted for by the temporary obstruction of the bronchia by the increased mucous secretion which usually accompanies this disease, and by the partial compression of the air-cells by those dilated. This supposition is corroborated by the fact, that persons affected with this disease have their breath much oppressed, in the first instance, when they chance to catch cold; while the respiration improves immediately after the expectoration commences, and even becomes better than before the catarrhal affection.—pp. 237–8.

This is a new disease, at least in practical medicine. It is only by the progress of pathological knowledge that we can hope for a true nosology. Under the term *Asthma*, as many very different diseases are confounded as under the term *Consumption*. It is hoped that the present work will be of no small use in leading to the discrimination of diseases, which cannot be confounded without the greatest injury to the subjects of them. Our author, unlike some of his countrymen, does not range all asthmatic affections as consequences of disease of the heart, although he evidently considers the disease as much more frequently symptomatic than idiophatic. If the term is to be retained in medicine, it ought to be restricted to the idiophatic or spasmodic variety; or used as the generic name comprehending the various species symptomatic of Bronchitis, Diseased Heart, Emphysema and Œdema of the Lungs, &c. as well as the nervous or the idiopathic, so well described by Dr. Bree. *Translator's note.*—p. 306.

### 2.—PERICARDITIS.

PERICARDITIS is inflammation of the serous membrane which lines the fibrous sac of the pericardium, the heart and large vessels. It may be either acute or chronic. This inflammation, like that of all membranes of the same kind, is marked by redness, more or less deep, a concrete albuminous exhalation and a sero-purulent effusion. The redness is almost always but slight in the acute disease. When it exists, it is for the most part only partially. It is most commonly punctuated, and looks as if the surface of the membrane was covered, here and there, with little specks of blood very close to each other. I have never perceived that this redness was accompanied by any thickening of the part. In some cases, wherein, to judge by the thickness of the false membranes, the inflammation appears to have been very great, no redness whatever can be discovered on the serous membrane, on removal of the membranous exudation. This concrete albuminous exudation commonly invests the whole surface of the pericardium, as well on the heart and large vessels, as on the sac. It rarely presents the appearance of an equable membranous layer, like the false membranes of pleurisy; on the contrary, its surface is most frequently marked by a great number of rough and irregular prominences. Sometimes the knobbed appearance of this exudation is very like what would result from the sudden separation of two pieces of slab joined by a pretty thick layer of butter; at other times, it is more like the internal surface of the second stomach of the calf, an observation made, in one case, by M. Corvisart. In certain cases this aspect of the false membrane has given rise to a singular error, having been mistaken for a variolous eruption in subjects dead of the small-pox. The consistence of this exudation is usually greater than that of the false membranes of pleurisy; it is also thicker, and more firmly adherent to the subjacent parts; its colour is, however, the same, being of a pale yellow anolagous to that of pus.

The serum effused in inflammation of the pericardium is limpid, of a pale yellow colour, or slightly brownish. It contains few fragments of semi-concrete albumen; at least, it very rarely contains enough of these to give it a milky and turbid character. The quantity of this effusion is usually considerable in the commencement of the disease, often as much as a pound. M. Corvisart found it, in one case, to amount to four pounds. It would seem that the quantity of effused serum diminishes quickly, as soon as the violence of the inflammation begins to subside; as we usually find the proportion of serum and albuminous exudation nearly equal, while in pleurisy and peritonitis the serum is commonly from twenty to fifty times greater than that of the extravasated lymph. Very commonly even, in very violent cases, we find no effused serum, and only a thick and highly concrete albumen filling the whole cavity of the pericardium, and uniting the heart and large vessels to the exterior or loose portion of this membrane. In this case we may suppose that the effused serum has been quickly absorbed, and the two layers of false membrane cemented together; although it is not impossible that, in some cases, the more solid exudation may be the only one. We have seen that the same thing occasionally takes place in certain partial and sub-acute inflammations of the pleura; and several observations have led me to believe that the cartilaginous patches that sometimes are met with on the exterior of the lungs (see page 51 and page 132) are produced in the same manner.

When the disease terminates favourably, pseudo-membranous exudation, after a certain time, is converted into cellular substance, or rather into laminæ of the same nature as the serous membranes; that is to say, the laminæ are double, the exterior surface being exhalent, and the interior cellular, or adherent, and containing the vessels distributed to the part. Sometimes these laminæ are long, sometimes so short that the pericardium seems intimately adherent to the heart.

Before the conversion of false membranes into cellular tissue was well understood, the adhesion of the pericardium to the heart was regarded by divers authors as a cause of various and serious complaints. Lancisi and Vieussens considered it as constantly causing palpitation; Meckel, as rendering the pulse habitually small; and Senac, as productive of frequent faintings. Even M. Corvisart himself has fallen into some mistakes on this head. He admits three species of adhesions,—all of which I have just described as mere varieties or stages of the same affection. These are, 1st, a demi-concrete albuminous adhesion, which is the only one recognised by him as the consequence of pericarditis; 2nd, the very intimate or close cellular adhesion, deemed an effect of gouty or rheumatic affections; and 3rd, the extended or long cellular adhesion, the cause of which is not assigned by him.* M. Corvisart is further of

*See Treatise on the Heart, &c. by M. Corvisart.

opinion that no person can live, and preserve a good state of health, who is affected with a complete and close adhesion of the pericardium to the heart, or of the lungs to the pleura.

I have, however, met with many cases where this condition of parts was found after death, in which no disorder of the respiration or circulation existed during life. A case adduced by M. Corvisart in support of his opinion (op. cit. p. 34) appears to me rather conclusive against it, inasmuch as the appearances on dissection showed sufficient lesions in other organs to account for the symptoms referred by him to the adhesions between the heart and pericardium.

Sometimes, though rarely, the inflammation is confined to a part only—sometimes a very small part—of the pericardium. These partial inflammations are in proportion to the general, in point of frequency, hardly as one to ten. Their anatomical characters are precisely the same, only that the albuminous exudation is in them confined to the inflamed part. The serous effusion is sometimes as abundant as in the general disease, more commonly, however it is less. The inflammation in this case almost always terminates in being cured, by the transformation of the pseudo-membranous exudation into long serous laminæ; scarcely ever are these partial inflammations followed by the intimate adhesion of the parts.

We frequently find on the surface of the heart opaque white patches, sometimes as large as the palm of the hand, more commonly one half or one third this size, and often very small. They are nearly of the thickness of the nail, and have a degree of consistence equal to that of the membranes composed of condensed cellular substance, as, for instance, the exterior membrane of the lymphatic glands. They adhere so closely to the parts on which they lie, that it is difficult to ascertain, even by dissection, whether they are situated above or beneath the fine membrane covering the heart and great vessels. M. Corvisart is of opinion that they are beneath it. I have, however, ascertained the incorrectness of this opinion, as I have several times been able to remove the patches, leaving the serous membrane of the pericardium still untouched.

Are these patches the effect of partial pericarditis and the consequent conversion of the effused lymph into a condensed membranous cellular tissue? M. Corvisart considers them as produced without previous inflammation, and seated, as I have already said, beneath the serous surface of the pericardium. Both these notions are, I think, inadmissible, inasmuch as there exists no example of an albuminous exudation on the adherent surface of a serous membrane, and as facts without number prove that pseudo-membranous exudations are always the produce of inflammation.

I have lately met with a case which appears to me to throw some light on the question of the origin of these spots. In a man dead of peripneumony, I found a thin false membrane, very firm and of a yellowish colour, investing the right auricle and a portion of the ventricle of the same side, all the rest of the pericardium being quite free, only containing in its cavity two or three ounces of a transparent and slightly yellow serum. Some parts of the false membrane, particularly on the auricle, were of a whiter colour and firmer than the rest, and exhibited an appearance almost the same as the white patches above described.

Chronic pericarditis is always general, occupying the whole internal surface of the serous membrane. This is commonly much redder than in the acute disease. The redness is formed by the close approximation of minute points, which look as if applied with a pencil. Rarely the chronic disease is accompanied by a pseudo-membranous exudation; and when this exists, it is thin, soft, friable, and entirely resembling a layer of very thick pus. In every case there exists a more or less copious effusion of a turbid, milky fluid, sometimes having quite a puriform character. I am led to believe that the close adhesion of the pericardium to the heart, is commonly the consequence of the absorption of this fluid, and that the adhesion by the long laminæ is the product of the acute disease. In one case I found a close and general adhesion of the pericardium to the heart and large vessels, by means of a false fibro-cartilaginous membrane, in every respect like those of the pleura.

From one case, cited by M. Corvisart, I am led to believe, that there may occasionally arise, subsequently to chronic inflammation of the pericardium, a tuberculous eruption similar to those frequently formed in the false membranes of the pleura and peritoneum. "The portion of the pericardium," he says, "which invests the heart, was of a greyish colour, thickened, unequal, wrinkled, crisp, and containing granulations of which the summit seemed ulcerated." I am rather led to consider these *granulations* as tubercles, because in the same subject "both lungs, although crepitous, were *granular* throughout." (Op. cit. obs. vii.)

In many cases of pericarditis, especially in the chronic disease, the muscular substance of the heart has lost its colour and become whitish. This loss of colour is sometimes attended by a notable degree of softening, and, at other times, the consistence is natural. Most writers have regarded this loss of colour as a mark of the inflammation of the heart itself, and most of the examples recorded of Carditis are merely cases of inflammation of the pericardium accompanied by this loss of colour. A great number of those collected by M. Corvisart are of this kind. For my own part I am disposed to doubt the correctness of the opinion that refers this loss of colour to inflammation. We can never be sure of the existence of inflammation in a muscular organ unless we find a deposition of pus among its fibres.—pp. 197—201.

1. *Acute Pericarditis.* There are few diseases attended by more variable symptoms, or of more difficult diagnosis, than this. Sometimes it appears with all the symptoms of a very violent disease of the chest; at others times it proves fatal without leading us, in the least, to suspect its existence. Again, we find cases marked by all the symptoms usually attributed by nosologists to this disease, and in the subjects of which, after death, we discover no traces of its existence. The same difficulty is acknowledged, or at least encountered, by most practitioners. Corvisart attributes the difficulty to the circumstance of pericarditis being almost "always complicated with pleurisy, peripneumony, or some other disease of the chest, which marks its peculiar symptoms." These complications, which are very common, must, unquestionably, have this effect where they exist; I must, however, confess, that the most completely latent affections of this kind that I have met with, were in subjects whose thoracic viscera were, in every other respect, quite sound, and who had died of disease of the abdomen. These facts seem to prove that inflammation of the pericardium is sometimes a local affection of little violence, and of very inconsiderable influence on the general system or even on the circulation; while, in other cases, it is accompanied by an acute fever, and by such violent disorder of almost all the functions, as to compromise the life of the patient.

M. Corvisart is likewise of opinion that it is when the disease is very acute, that the symptoms are very obscure. "Its invasion," he says, "is sudden, its progress rapid, its termination almost instantaneous." When it exists in a less violent degree, but still acute, he thinks that it can be recognised by the following symptoms: viz. sense of heat in the region of the heart; great difficulty of respiration; greater colour of the left cheek than the right; pulse at first frequent, hard, and rarely irregular,—becoming about the third or fourth day, small, hard, contracted and often irregular; great anxiety, slight palpitations, partial faintings; *peculiar* change of features; and (towards the close of the disease) total or partial cessation of the local pain.

These symptoms are certainly sometimes present in pericarditis; but each, or all of them may be absent, and some of them are very rare. I have never observed the increased colour of the cheek, have rarely heard complaints of local heat or pain, and, in place of the progressive increase of irregularity in the pulse (as described by M. Corvisart,) I have uniformly found this irregularly intermitting, wiry, and almost insensible, from the very commencement of the disease.

I must admit that the stethoscope scarcely furnishes us with any more certain signs of this disease. The following appear to me to be the most common symptoms of the inflammation of the pericardium, when not latent: the contraction of the ventricles yields a greater shock, and sometimes a more marked sound, than usual, and, at intervals, feebler and shorter pulsations are perceived, which correspond with intermissions of the pulse, the smallness of which contrasts remarkably with the strength of the heart's pulsation.— When these symptoms come on suddenly in a person who had never been affected with disease of the heart, there is great probability of their being the consequence of this disease. In addition, it is further common for the patient to have much dyspnœa and very great anxiety; and to suffer syncope on taking a few steps, or on moving suddenly in his bed.

2. *Chronic Pericarditis.* The signs of this variety are still more uncertain than those of the acute disease. I have attended several cases which I considered, throughout their whole course, as chronic inflammations of the pericardium, but which almost all were cured. In two or three cases only have I been able to verify the correctness of my diagnosis by examination after death; whilst very frequently I have found the pericardium full of pus and in a true state of chronic inflammation, without having been at all led to suspect such an affection. In the cases which have occurred within the last three years, I have found the symptoms to be precisely the same as in the acute disease, only less violent. From one to two years has elapsed before a cure has taken place; and when this has been effected the action of the heart and pulse has become natural and regular.–pp. 273–375.

### 3.—SOFTENING OF THE HEART.

This enlargement of the heart is always attended by a considerable increase of its consistence, except when conjoined with another affection of this organ, to be noticed presently, viz: *softening of the heart.*—p. 165.

I have already noticed this condition of the heart. In it the muscular substance is sometimes so soft as to be almost friable, the fingers passing easily through the parietes of the ventricles. Whatever may have been the patient's disease, the heart is rarely filled with blood, and the ventricles equally collapse whatsoever may be their varying thickness. This affection of the heart is almost always attended by some change of colour in the organ. Sometimes this is deeper, and even quite violent; and this is particularly the case in fevers of the kind named *adynamique* by Pinel. More commonly, however, the softening of the heart is attended by a striking loss of colour, so as to resemble the palest dead leaf. This pale or yellowish tint does not always occupy the whole thickness of the heart; sometimes it is strongly marked in the central portions, and very little on the exterior or inferior surfaces. Frequently the left ventricle and interventricular septum exhibit this appearance, while the right ventricle retains its natural colour, and even a degree of firmness greater than natural. Again, we sometimes find here and there spots of the natural colour and consistence in hearts which are, every where else, much softened and quite yellowish. This variety of yellowish softening is particularly observable in those cases where dilatation is conjoined with a slight degree of thickening. It is also found in simple dilatation, although it is more common to find this state accompanied by that species of softening which is marked by an augmentation of the natural colour of the organ. There is a third variety of softening of the heart, which will be noticed in another place, and which is attended by a pale white colour of the muscular substance. In this, the degree of softening never reaches that of friableness; often it is scarcely perceptible; but the parts are flabby, and the parietes of the ventricles quite fall together on being opened. This condition will be noticed under the head of inflammation of the pericardium, as it is peculiar to that disease.

It would seem that the softening of the heart discovered in subjects whose death has been very gradual, is an acute affection; it is evidently still more so where it exists only partially in the substance of the organ. On the contrary, in cases where the heart is softened and yellowish throughout, it is probable that the affection has existed for a long time. The deep-coloured softness observed in subjects dead of fever, may, I think, be compared to that adhesive softness of the other muscles often observed in these cases, and which is also accompanied by a degree of redness greater than natural. This softening of the heart, as well as the analogous *gluey* or *fishy* (gluant ou poisseux) state of the muscles, is particularly observable in putrid fevers, particularly when these exhibit the phenomena formerly considered as marks of putridity—viz: livid intumescence of the face, softening of the lips, gums, and internal membrane of the mouth, black coating on the tongue and gums, earthly aspect of the skin, distended abdomen and very fetid dejections. I cannot assert that this softening of the heart exists in all kinds of continued fevers, but I have met with it constantly in such cases as I have attended to. Could it account for that frequency of pulse which exists, sometimes for several weeks, in convalescence from fevers, although the patient continues to regain flesh and vigor?—pp. 171–2.

Cases of total softening of the heart are usually accompanied by a certain degree of cachexy, even when the individuals are otherwise in tolerable health. When such subjects are attacked with dilatation or hypertrophia of the heart, as almost always happens, they do not present the usual swollen and livid state of the face observable in other cases of this sort.

When softening exists along with dilatation of the ventricles, the sound produced by the contraction of these cavities, although loud, is yet dull, and without the clearness which attends common dilatation. When it is complicated with hypertrophia, the sound of the contraction of the ventricles is so obtuse as to be nearly inaudible; and in extreme cases, the impulse of the heart is attended by no noise whatever.—p. 271.

